# Case of Carbuncle

**Published:** 1852-11

**Authors:** Clinton Colegrove


ART. IV.—*Case of Carbuncle.* Reported by CLINTON COLEGROVE, M. D.

April 18, 1852. Was called to see Mr. R. T., aged 71, temperament sanguine, and previous health good. Had, last February, wandering pains in his back and neck, followed by constant and excessive itching. On the 3d instant, a slight swelling was noticed along the back of the neck, about the junction of the cervical and dorsal vertebræ, which was accompanied by great pain and heat, and which rapidly became diffused over a large extent of surface measuring from eight to twelve inches in diameter, and reaching nearly to the head, purple and projecting outward from the base nearly four inches.

We noticed numerous small orifices which covered the face of the tumor, from which oozes a thin, purulent, sanguineous matter. The tumor is quite hard and seems to be of dense structure, communicating an impression to the finger like that of dry sponge.

Of the treatment heretofore I know nothing, it having been conducted by a *Root Doctor.*

We made a conical incision into the tumor, each cut being about two and a half inches in length, and applied a charcoal poultice, with liberal internal use of opium and Port wine.

April 29. Found our patient restless, particularly nights. Pulse 100 and feeble. Applied the knife more deeply in the line of the original incisions, which bled freely. The subcutaneous cellular tissue is cut with difficulty, and seems to have acquired a kind of cartilaginous hardness. The treatment consists in the application of a wine poultice. Internally free administration of carb. ammonia and opium—of the former, ℈jss, of the latter, gr. vi, dissolved in an ounce and a half of water. Dose, ʒi. every fourth hour.

May 9. Patient decidedly worse. The edges of the incisions made at the last visits, are gangrenous, and the whole aspect of the tumor is livid. Large portions of the cellular tissue are sloughing, accompanied by a fœtid purulent discharge. Both feet, left hand and arm œdematous. Pulse 120, very feeble. Respiration somewhat hurried and laborious. General appearance languid and sunken. Has sharp lancinating pains extending through the tumor and upper part of the chest. Prescribed, opium grs. ii, every eighth hour. Port wine freely, and a solution of cream of tartar for a drink.

May 12. Very extensive sloughing of the cellular membrane, and excessive œdema of the feet, left hand and arm, with slightly erysipelatous condition of the skin. Applied a broad bandage to the feet and ankles, to be kept wet with cold water. Above treatment continued.

May 15. The sloughing has continued rapidly and obstinately. The bandages gave pain and were removed.

May 18. Patient seems on the whole to be better. The sloughing has continued so that the introduction of a probe under the edges discloses a large sulcus, two inches in diameter, around the whole circumference of the ulcer.

We now commenced the administration of tonic medicines, as large doses of quinine, &c. Also some diuretics.

May 23. Patient better. Sloughing has entirely discontinued.

May 29. Patient improving. The integument is slightly attached to the surface underneath. Adhesive straps were applied with a view to accelerate an approximation of the lips of the ulcer.

Aug. 15. Called on my old patient to-day, and am gratified to find him in excellent spirits and health, with the single exception of the sore upon his back, which, under a continuation of the treatment last adopted, has become smaller and smaller, and is now about one inch in diameter, with every prospect that it will soon be perfectly healed.

